# Statewide Universal Newborn Screening for Human Immunodeficiency Virus

**DOI:** 10.3390/ijns12040081

**Published:** 2026-10-02

**Authors:** Norma P. Tavakoli, Melissa Pearce, Richard Sejour, Daniele-Marisa Stansfield, Marilyn Erickson, Rebecca McMahon

**Affiliations:** 1 Division of Genetics, Wadsworth Center, New York State Department of Health, Albany, NY 12208, USA; melissa.pearce@health.ny.gov (M.P.); richard.sejour@health.ny.gov (R.S.); daniele-marisa.stansfield@health.ny.gov (D.-M.S.); marilyn.erickson@health.ny.gov (M.E.);; 2 Department of Biomedical Sciences, State University of New York, Albany, NY 12208, USA

**Keywords:** human immunodeficiency virus, ELISA, newborn screening, mother-to-child-transmission

## Abstract

Universal newborn screening for human immunodeficiency virus (HIV) has been mandatory in New York State (NYS) since 1997. The goal of screening is to prevent mother-to-child transmission (MTCT) by using approaches including antiretroviral therapy (ART) in the newborn and lactating mother. Screening is performed using an enzyme-linked immunosorbent assay that detects maternal antibodies in dried blood spots from newborns. From January 2011 through November 2018 and from October 2022 through December 2024, the NYS Newborn Screening Program used the HIV-1 MicroELISA System to screen for anti-HIV-1 antibodies in these samples. Newborns who screened positive were referred to an infectious disease specialist for follow-up. During the period of this study, approximately 2.3 million newborns were screened for HIV-1 antibodies. Amongst 4018 newborns referred for follow-up, MTCT was confirmed in 16 newborns (0.4%), 3930 were uninfected (97.8%), 53 were lost to follow-up or were deceased (1.3%), and 19 newborns were considered to have false-positive HIV results (0.5%). MTCT occurred in one in 144,162 newborns screened during the study period, while one in 577 infants were considered to be at risk of vertical HIV transmission. The numbers of newborns who screened positive for HIV antibodies and were therefore at risk of MTCT as well as MTCT have both fallen from 2011 to 2024. NYS MTCT has been below 1% since 2015. To further reduce transmission risk, factors that contribute to loss to follow-up of infants exposed to HIV should be investigated.

## 1. Introduction

Perinatal human immunodeficiency virus (HIV) transmission can occur during pregnancy, childbirth, or breastfeeding and is the main cause of infection in pediatric cases [1]. Delays in diagnosis and treatment of HIV-infected newborns can lead to significant morbidity and mortality, especially as newborns are at a greater risk of opportunistic infections because their immune system is not fully developed.

In the US, acquired perinatal HIV cases peaked in 1992 with an estimated 1650 cases and an incidence of 43.1 per 100,000 live births [2,3]. At the time, mother-to-child transmission (MTCT) rates were estimated to range from 14 to 25% in developed countries and nearly twice as high in Africa [4]. In New York State (NYS), from November 1987 to November 1988, the overall HIV seroprevalence from anonymous newborn screening data was 0.66% (1816 newborns), and it was estimated that approximately 40% of that cohort would become infected [5]. In 2012, the Centers for Disease Control and Prevention (CDC) published a *Framework for Elimination of Perinatal Transmission of HIV in the United States* with the goal of reducing the rate of perinatal transmission to <1% [6]. Strategies such as early diagnosis using comprehensive serologic screening and nucleic acid tests, treatment of HIV-infected pregnant women, prophylaxis and treatment of at-risk newborns using antiretroviral therapies (ART), and, when appropriate, elective cesarean section and avoidance of breastfeeding have helped reduce the risk of MTCT [7,8,9,10,11]. Published data indicate such approaches can reduce the rate of MTCT to <1% [12,13,14].

Between 1988 and 1994, for surveillance purposes, most states in the US anonymously screened dried blood spot (DBS) specimens for HIV in a program called the Survey of Childbearing Women [15]. After effective treatment to reduce transmission risk became available, both the CDC and the American Academy of Pediatrics (AAP) recommended universal voluntary testing of pregnant women [16,17]. Additionally, several states such as Texas, New Jersey, Connecticut, and Illinois implemented perinatal HIV testing laws specific to newborn testing, including testing newborns for HIV if the mother’s HIV status is unknown [18]. Currently, as part of standard prenatal care, pregnant women are screened for HIV at least once during their pregnancy and frequently also in the third trimester [19,20]. In NYS, mandatory newborn screening for HIV began in February 1997 [21,22], and NYS remains the only US State where all DBS specimens submitted to the Newborn Screening (NBS) Program are screened for HIV. The goal of screening newborns is to detect cases in which the mother’s HIV status is unknown or in cases where the mother was infected late in pregnancy and has not yet been identified as HIV-positive. Identification of cases depends on whether detectable antibodies are present, which may be influenced by factors including timing of infection, mother’s viral load, and suppressive maternal ART.

NYS has a population of approximately 20 million and between 2011 and 2024 had an annual birth rate of between 204,000 and 241,000 live births [23]. Since 2011, the NYS NBS Program has mainly used the HIV-1 MicroELISA System (Avioq, Inc., Durham, NC), an enzyme-linked immunosorbent assay (ELISA), to screen newborns for HIV-1. The kit is approved by the US Food and Drug Administration (FDA) for detecting HIV-1 in DBS among other specimen types, which is a major reason for its adoption by the NBS Program. The assay is a second-generation HIV test and detects antibodies (IgM/IgG) to HIV-1 and may also detect HIV-2 antibodies that cross-react with HIV-1 viral lysate proteins, which are the primary antigens used in the test. Serological testing of blood eluted from DBS specimens assesses the HIV status of mothers because maternal antibodies passively transmit across the placenta and remain present in the infant for months after birth. Nucleic acid testing (NAT) technologies (e.g., RNA amplification assays) must be used to determine if the infant is HIV-infected [24]. Testing should be performed at birth and also at various subsequent times depending on the infant’s risk category [25].

Here, we present data for screening NYS newborns for HIV-1 antibodies in DBS using the HIV-1 MicroELISA System. As part of short-term follow-up of referred newborns, providers were required to perform NAT and relay the results to the NBS Program.

## 2. Materials and Methods

The NYS NBS Program requested that DBS specimens be collected at 24–48 h of life prior to 13 February 2019 and between 24 and 36 h after that date. Specimens were mailed to the screening laboratory overnight at ambient temperature. The newborns’ demographic information, including sex, birth weight, gestational age, race/ethnicity, and mothers’ HIV testing status, was also submitted. After 2012, race/ethnicity data collection was suspended due to its subjective nature. All specimens were screened for the NYS panel of analytes, which included HIV antibodies, as long as there was sufficient specimen available for testing. The HIV-1 MicroELISA System was used to screen newborns for HIV-1 from 1 January 2011 to 30 November 2018 and from 1 October 2022 to 31 December 2024 for a total of 10 years and 2 months. An alternative methodology to detect HIV-1 antibodies was used from December 2018 through September 2022. Results of HIV-1 screening from that period in NYS are excluded from this report.

The HIV-1 MicroELISA System includes both liquid positive control and liquid negative calibrators, which were deposited directly into MicroELISA plate wells. Additionally, DBS quality control material available from the CDC was included on each 96-well plate. The assay protocol used was essentially the same as the manufacturer’s (Avioq) protocol. However, to improve the workflow in the laboratory, several minor modifications were made to the kit protocol: a single 3.2 mm DBS punch per baby was eluted overnight, or up to 3 days, at 2–8 °C using 165 µL of DBS elution medium. Seventy-five µl of Dilsim™ III was added to each well of the MicroELISA coated plate, and 75 µL of eluate was added to each corresponding well. Plates were incubated at 37 °C for 90 min, then washed 4 times with wash solution, with a 30 s soak in between washes. One hundred and fifty microliters of reconstituted peroxidase-conjugated goat anti-human immunoglobulin were dispensed in each well, and plates were incubated at 37 °C for 40 min. The wash step was repeated, followed by 12 min incubation at room temperature with 150 µL of 2,2′-azino-di-[3-ethylbenzthiazoline-6-sulfonate] substrate solution. The reaction was stopped with 150 µL of sodium fluoride stop solution, and the absorbance of each well was measured at 405 nm. The HIV-1 MicroELISA System is FDA-approved, but because of the minor modifications to the assay, per regulatory requirements in NYS, prior to clinical use, validation studies were performed and submitted to the NYS Clinical Laboratory Evaluation Program (CLEP), and the assay was approved locally.

Per manufacturer’s instructions, if assay controls and calibrators were within acceptable limits, assays were considered valid. Any specimen with a signal-to-cutoff (S/CO; optical density value of the test sample divided by optical density value of the assay’s cutoff) value of ≥1.0 was considered positive for HIV and was retested in duplicate. DBS specimens from newborns whose mothers were known to be HIV-positive were also retested in duplicate for HIV, irrespective of the initial HIV result. These newborns were referred for follow-up to the hospital of birth and an infectious disease specialty care center if the HIV result was confirmed by retesting. If the mother was known to be HIV-positive, the recommendation is for the newborn to be tested by NAT within 48 h of birth and referred to a pediatric infectious disease specialist for further monitoring/testing. If the mother’s HIV status was unknown at birth, expedited testing was offered to the mother, and if declined, the newborn was tested at delivery. Expedited testing was arranged by the hospital of birth and frequently performed on-site. If the mother had tested negative during pregnancy, but the newborn screened positive by NBS, then both mother and newborn were tested. If results indicated that the mother had seroconverted, then both mother and newborn were referred for additional testing/treatment/prophylaxis. In all cases where NAT of the newborn was performed, the NBS Program closed out cases when it received information on the initial NAT result, as this confirmed that the newborn was being followed. The NBS Program shared screening results with the NYS AIDS Institute, which is responsible for ensuring that HIV-exposed infants receive appropriate and timely diagnostic testing and care.

Newborns who were screen-positive and whose mothers were known to be HIV-positive are referred to as *confirmed screen-positive,* and those whose mothers were screen-negative and whose NAT results were negative are referred to as *false-positive screens*. Newborns who were screen-positive and NAT-positive are referred to as *infected with HIV*.

### Statistical Analysis

All statistical analysis was performed using the R Statistical Computing software (version 4.5.1) [26]. Two-tailed Fisher’s exact tests were calculated to determine the association between newborns determined by newborn HIV screening to be potentially at risk of HIV infection (HIV screen-positive excluding false-positive screens) versus those not at risk (HIV screen-negative and false-positive screens) and their specific demographics (e.g., sex, birth weight, gestational age, etc.). Fisher *p*-values were estimated through Monte Carlo simulations with 1,000,000 replicates. The odds ratios were calculated with 95% confidence intervals. For each table, *p*-values were corrected for multiple comparisons using the Holm–Bonferroni method. A *p*-value < 0.05 was considered statistically significant.

Regression statistics were conducted using the mean HIV immunoassay ratios as the response variable, and the explanatory variable was the HIV status of various groups. Nonparametric statistics were performed on this model because regression diagnostics revealed profound non-normality among each group. For omnibus statistics, the Kruskal–Wallis test was conducted. For pairwise analyses between the HIV status groups, the Wilcoxon Rank-Sum Test was used, and *p*-values were adjusted using the Holm–Bonferroni method.

HIV exposure rates per county were calculated using the CDC WONDER dataset issued from 2011 to 2024 [23]. The data was collected by the National Center for Health Statistics and was derived from birth certificates.

## 3. Results

### 3.1. Outcome of HIV Referrals

During the 10-year and 2-month period when the HIV-1 MicroELISA System was used for HIV-1 screening in NYS, 2.3 million newborns (2.6 million specimens) were screened, and 4018 were referred for HIV follow-up (Table 1). Due to a steady decline in birth rate, the number of newborns whose specimens were submitted to the NYS NBS Program fell by 12.5% between 2011 and 2024. In addition, the number of newborns with screen-positive results who were referred for HIV follow-up also fell from a high of 479 in 2011 (202 per 100,000; born to 459 mothers) to 277 in 2024 (134 per 100,000; born to 264 mothers) (Table 2). Of the newborns with screen-positive results, between 0 and 6 cases of MTCT were recorded each year, totaling 16 newborns infected with HIV (Table 2). MTCT fell from 2.5 per 100,000 in 2011 to 0.48 per 100,000 in 2024. Since 2015, the rate of MTCT has remained below 1 in 100,000 live births (including from December 2018 through September 2022).

Of the 16 newborns who were infected with HIV, 10 had mothers known to be HIV-positive and were being followed. Of the remaining six, one mother was tested a month prior to delivery and tested negative for HIV. In a second case, the mother was tested seven months and also three weeks prior to delivery and both times tested negative for HIV. Expedited HIV testing was performed at birth in the four remaining cases because the HIV status of the mothers was unknown. Two of the four mothers were reported not to have had HIV testing performed during pregnancy.

**Figure 1:**
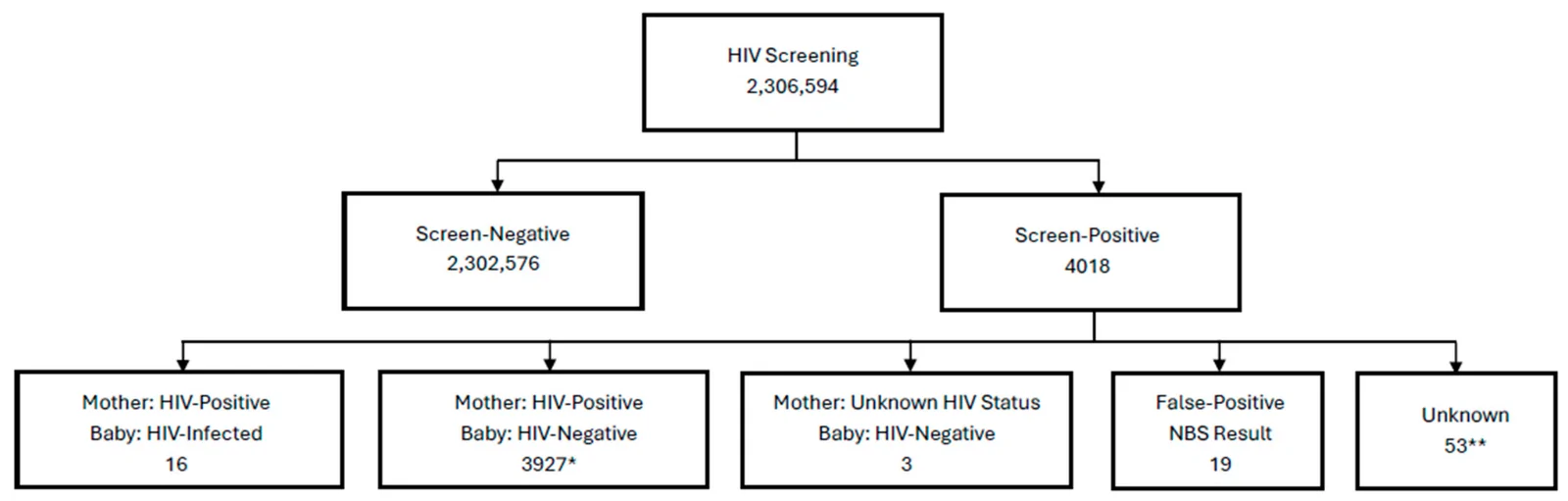
Outcomes of screening for HIV using the MicroELISA System. * Three thousand nine hundred and twenty-two newborns had mothers who were positive for HIV-1, and five had mothers who were positive for HIV-2, including one deceased newborn. ** Includes 43 newborns who were lost to follow-up, one newborn whose parent declined follow-up, and 9 deceased newborns. Mothers of 45 of 53 newborns were reported as HIV-positive. Mothers of the remaining 8 newborns were reported as HIV-negative (*n* = 6), HIV status unknown with expedited testing performed at birth (*n* = 1), and information is missing (*n* = 1). HIV, human immunodeficiency virus; NBS, newborn screen.

**Figure 2:**
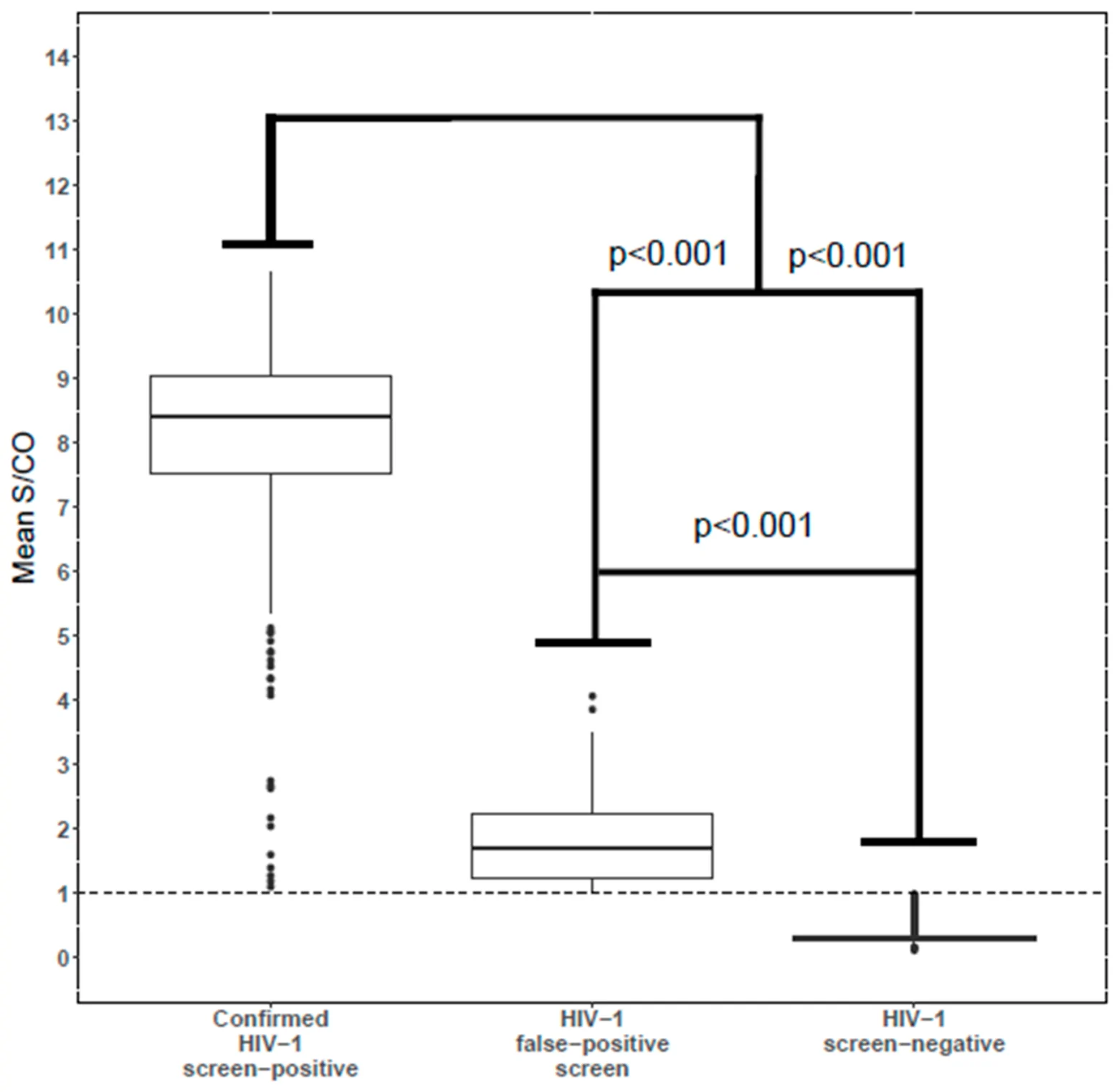
Comparison of S/CO values for newborns who were non-reactive, confirmed with HIV antibodies, and false-positive for HIV antibodies. The average S/CO ratios for all specimens were graphed as boxplots for the following three categories: confirmed HIV-1 screen-positive cases from 2024 (*n* = 258, mean S/CO = 7.98, median S/CO = 8.42, and SD = 1.76), HIV-1 false-positive cases for the entire period of the study (*n* = 19, mean SC/O = 1.98, median S/CO = 1.72, and SD = 0.98), and HIV screen-negative cases from 2024 (*n* = 206,790, mean S/CO = 0.3, median S/CO = 0.29, and SD = 0.047). Outliers were determined as values greater than or less than 1.5× the interquartile range. The *p*-values were calculated using the nonparametric Wilcoxon Rank-Sum Test and were corrected using the Holm–Bonferroni method. A *p*-value < 0.05 is considered statistically significant. The dots represent outliers and the dashed line represents the cutoff for the assay (mean S/CO ≥ 1.0 was considered screen-positive for HIV). S/CO, signal-to-cutoff ratio; HIV, human immunodeficiency virus; SD, standard deviation.

Amongst 4018 newborns with screen-positive results referred for HIV follow-up, the large majority (*n* = 3930; 97.8%) were not infected with HIV while their mothers were infected with HIV-1 (*n* = 3922), with HIV-2 (*n* = 5), or their status was unknown (*n* = 3) (Table 2, Figure 1). Additionally, in 53 cases (1.3%), the newborns were lost to follow-up (*n* = 44) or were deceased (*n* = 9), and in 19 cases (0.5%), the NBS result was considered to be false-positive (Table 2). In the latter 19 cases, diagnostic follow-up data indicated that both mother and baby were HIV-negative. The average ELISA S/CO value for newborns with false-positive results ranged from 1.02 to 4.07, with a mean of 1.98, compared to a S/CO mean of 7.98 for the confirmed positive cases in 2024. This indicates that compared to the confirmed positive cases, the false-positive cases had an S/CO value closer to the assay cutoff of 1.0 (Figure 2). In addition, when we sought the HIV results of siblings before 2011 or after 2024 for the 19 newborns with false-positive results, we found that three had siblings who also had a false-positive HIV NBS result.

From 2011 to 2024, excluding the period from December 2018 through September 2022 when an alternative testing methodology was used, and excluding 19 cases with false-positive results, a total of 3999 (0.2%) newborns were potentially exposed to perinatal HIV based on positive NBS results (Table 2). This number includes 175 newborns (87 sets of twins and 1 set of triplets) from multiple-birth pregnancies, two of whom had twins who passed soon after birth and were not screened for HIV. A total of 3824 single births had potential perinatal exposure to HIV. The 3999 newborns were born to 3161 mothers. NBS cards from an additional 982 newborns indicated the mothers’ status as HIV-positive, but the HIV newborn screen was negative.

### 3.2. Mothers’ HIV Status

Based on the mothers’ HIV status as reported on the NBS cards, in a large majority of pregnancies in which newborns screened positive and were potentially exposed to HIV, the mothers were known to be HIV-positive (3667/3999; 91.7%). For the remaining 332 cases, the NBS Program attempted to clarify the HIV status of the mothers and determined that an additional 227 mothers were also known to be HIV-infected, but the NBS forms were incorrectly completed. In 92 (2.3%) cases, the mothers’ HIV status was unknown or missing, and in 13 (0.3%) cases the mothers had tested negative during pregnancy but had seroconverted prior to giving birth. Expedited testing was performed at birth for 141,887 mothers (6.2% of births), 394 (9.9% of cases with screen-positive results excluding those with false-positive results) of whom had newborns with a positive HIV newborn screen. The number (%) of mothers reported to the NBS Program as having unknown HIV status fell from 6419 (2.7%) in 2011 to 2241 (1.1%) in 2024.

### 3.3. Characteristics of Newborns at Risk of HIV

There was no significant difference between the numbers of male and female newborns who were potentially exposed to HIV (Table 1). However, compared to the general population, a larger proportion of newborns exposed to HIV had lower birth weight, were preterm, and required care in the neonatal intensive care unit (NICU) (*p* < 0.001; Table 1). Race/ethnicity data is not available for newborns born in recent years, as the NBS Program did not request this data after 2012. Of the 927 newborns potentially exposed to HIV during 2011 and 2012, 480 (51.8%) were reported to the NBS Program as Black, 191 (20.6%) were reported as Hispanic, 146 (15.7%) were reported as White, and 14 (1.5%) were reported as Asian (Table 1). Of the seven newborns who were positive by NAT in 2011 and 2012, three were reported as Hispanic, three as white and one as Black. From 2011 to 2012, the number of NBS HIV-positive White newborns decreased from 92 to 36 per 100,000 births, whereas the number of Black/African American newborns increased from 563 to 672 per 100,000 and the number of Hispanic newborns from 225 to 246 per 100,000. During those 2 years, the number of NBS HIV-positive Asian newborns decreased from 54 to 19 per 100,000 births.

Based on NBS results during the study period, the majority of newborns potentially exposed to HIV lived in New York City (NYC), with the highest numbers in the boroughs of the Bronx (*n* = 1250; approximately 609 per 100,000 live hospital births) and Kings County (*n* = 878; approximately 220 per 100,000 live hospital births). Queens County, which, after Kings County, has the highest birth rate of the five NYC boroughs, had a lower exposure rate (*n* = 410; approximately 141 per 100,000 live births). Not including NYC and Long Island, significant numbers were recorded in Erie (*n* = 129; approximately 131 per 100,000 live hospital births) and Monroe (*n* = 80; approximately 99 per 100,000 live hospital births) Counties. Nineteen counties and boroughs in NYS had 15 or more newborns at risk of HIV during the period of this study.

## 4. Discussion

In the US, the estimated incidence of perinatally acquired HIV infection at its peak in 1992 was 43.1 per 100,000 [3]. In NYS, the incidence has been below 1 per 100,000 births since 2015. Perinatal HIV transmission fell significantly from 11% in 1997, when NBS for HIV was first implemented by the NYS NBS Program [21], reaching the CDC goal of <1% by 2012, where it has remained through 2024.

From 2011 to 2024, the number of newborns in whom HIV antibodies were detected by NBS fell from 481 to 277 (by 43%), and the number of MTCT fell from six cases in 2011 to one in 2024. Most of the newborns referred for HIV follow-up had mothers who were known to be HIV-positive and were therefore being followed. However, the status of a small number (*n* = 6) was unknown, and it is the detection of these newborns through NBS that has the greatest impact. When the mother’s HIV status is unknown, the newborn must have expedited HIV testing performed [11]. It is especially important that the NBS Program detects HIV exposure in these newborns in order to initiate follow-up, including diagnostic testing and administration of ART. Appropriate ART administration is recommended as soon as possible after birth and preferably within six to 12 h after delivery [27]. Information on the mother’s HIV status is therefore critical in order to initiate ART in the appropriate timeframe. NBS test results, however, are not available for several days after birth, which is not optimal for initiating therapy in a timely manner but is preferable to any further delay in diagnosis and treatment of both mothers and newborns detected by NBS.

During the 10-year and 2-month period in which the HIV-1 MicroELISA System was used for HIV NBS, 19 false-positive results were reported by the NBS Program. In these cases, diagnostic testing indicated that both mother and newborn were negative for HIV. This is a false-positive rate of 0.001%. Other second-generation HIV ELISAs have been reported to have false-positive rates of <1%, with one study reporting specific values of 0.02% and 0.17% for two different second-generation screening assays when screening fresh sequential blood donations [28,29]. Generally, false-positive results are due to cross-reaction of the test’s antigen component or antibody cross-reactivity. In the majority of the 19 cases with false-positive results, the S/CO values were close to the cutoff, indicating a low positive value (Figure 2). Interestingly, in three of 19 cases, siblings also had false-positive NBS results for HIV, indicating consistent presence of proteins in the mothers that cross-react with HIV antigens. Specific factors such as infection, vaccination, tumor, autoimmune diseases, heterophile antibodies and pregnancy and non-specific factors such as hemolysis, hyperbilirubinemia and pH value can lead to false-positive results [30,31,32,33,34,35,36]. There have also been recent reports of increased false-positive HIV test results in individuals with acute or past severe acute respiratory coronavirus 2 (SARS CoV-2) infection [37,38,39]. Interestingly, 10 of the 19 false-positive cases were detected in the years after the coronavirus disease 2019 (COVID-19) pandemic, although we are unable to draw conclusions as the SARS CoV-2 status of the mothers was not reported to the NBS Program. False-positive ELISA results demonstrate the importance of verifying ELISA results with a more specific confirmatory test.

Of the 4018 newborns who were referred for HIV follow-up, 44 (1.1%) were lost to follow-up. Travel out of state as well as societal issues such as poverty, mental health challenges, substance-use disorder, fear of stigma and discrimination, and barriers to health care access are possible reasons for loss to follow-up [40,41]. Investigating causes of follow-up loss and eliminating gaps in care for these mothers and newborns will help to further reduce the risk of MTCT.

Data from our study indicate that women living with HIV have an increased risk of preterm birth and giving birth to newborns with low birthweight (LBW), compared to women who are HIV-negative, as has been previously reported (Table 1) [42,43]. In addition, our data indicates a higher probability that mothers who are HIV-positive give birth to newborns who require care in the NICU (Table 1). Newborns born to mothers who are HIV-positive are at risk of outcomes such as stillbirth, admission to the NICU, congenital anomalies, higher neonatal anomalies, and death [44,45,46]. Early infant ART initiation is associated with viral suppression and restricting the viral reservoir size, consequently significantly reducing infant morbidity and mortality [46,47,48].

In NYS, most newborns at risk for HIV lived in the NYC boroughs of the Bronx and Kings County, both of which have diverse populations and high poverty rates [49,50,51]. NBS data from 2011 and 2012, when race/ethnicity information was collected, indicate that Black and Hispanic newborns were disproportionately affected by HIV, as has been reported previously [14]. During those 2 years, Black/African American newborns were 17.1 times more likely and Hispanic newborns were 6.5 times more likely than Asian newborns to be perinatally infected with HIV. The race/ethnicity disparities had declined by 2019 but have continued to persist [14].

As of 2018, it was estimated that in the US the number of HIV-positive women who gave birth was approximately 5000 annually, the majority of whom would have had an HIV diagnosis before pregnancy [52]. However, up to 30% of mothers of infants infected with HIV are unaware of their HIV status until or after delivery [53]. In order to reduce MTCT, it is important to perform early diagnosis, link women of reproductive age/pregnant women to care, initiate early ART, and ensure adherence. Furthermore, retesting mothers who are at increased risk of acquiring HIV during the third trimester, in addition to the first trimester, could help identify mothers who acquire HIV later in pregnancy [54].

During the period of this study, in NYS there was a general decline in the number of HIV-infected women giving birth from 459 in 2011 to 264 in 2024. Thus, the number of women giving birth who were infected with HIV fell by 42.5% from 2011 to 2024, while the birth rate only fell by 12.5%. Of 3999 newborns potentially exposed to HIV during the study period, 92 had mothers whose HIV status was unknown or missing from the NBS cards. An additional 982 newborns were HIV screen-negative but had mothers who were reported as HIV-positive on the NBS cards. It is possible that in some of these cases the antibody levels were too low to be detected by NBS and in other cases the NBS cards could have incorrect information regarding the mothers’ HIV status. Irrespective of NBS results, newborns born to HIV-positive mothers should receive ARV prophylaxis shortly after birth, a diagnostic test (NAT) within 48 h of birth and should be referred to a pediatric infectious disease specialist for further follow-up [11,25].

Worldwide, most HIV infections are caused by HIV-1. HIV-2 is endemic in West Africa, although cases have also been described in other countries, including the US [55,56,57]. Between 2011 and 2016, NBS detected HIV antibodies in five newborns whose mothers were infected with HIV-2, and yet the MicroELISA System detected those cases despite targeting HIV-1 antibodies. Detection of HIV-2 was likely due to the cross-reaction between antibodies to HIV-2 and the total HIV-1 lysate proteins coated on the MicroELISA test plates.

The use of the MicroELISA System for NBS had several advantages. The kit was FDA-approved for use with DBS and allowed high-throughput testing, as it was available in 96-well format. The turnaround time for obtaining negative results was generally two business days, and for positive results was three business days after specimen receipt by the NBS Program. The test is simple to perform, relatively inexpensive, and does not require complex instrumentation. The false-positive rate for the screen was 0.001%, and we are not aware of any false-negative results for any newborn who seroconverted during the period this screening test was used.

### Limitations of the Study

Limitations of the study included a non-standardized collection of race/ethnicity data between 2011 and 2012, lack of race/ethnicity data after 2012, lack of data for cases that were lost to follow-up, and occasional incomplete or incorrect demographic data. For example, there was missing or incorrect information regarding sex, birth weight, gestational age or mother’s HIV status. In cases when the NBS Program detected a positive HIV result, but the NBS card indicated that the mother was not infected with HIV, the NBS Program contacted the hospital of birth to clarify the mother’s HIV status. Frequently, the form was incorrectly completed. Other limitations included the lack of a cost-effectiveness analysis for newborn screening for HIV in NYS.

## 5. Conclusions

Our data indicate that universal NBS for HIV has been successful in acting as a safety net to detect newborns at risk of HIV whose mothers have an unknown diagnosis. The multipronged approach of screening women during pregnancy, screening the mother and/or baby at the hospital of birth when the mother has an unknown diagnosis, and newborn screening for HIV minimizes the risk of missing newborns at risk of MTCT. The MicroELISA HIV-1 System was a suitable testing method in a high-throughput NBS setting using DBS specimens. We also show that the implementation of strategies to reduce MTCT has been successful in NYS because, since 2015, the incidence of perinatally acquired HIV has been <1 per 100,000 live births, which was the goal set by the CDC in 2012.

## Figures and Tables

**Table 1:** Screening results and newborn demographic information during study period.

Characteristics	Total Screened (%)	Referred (%)	Potentially at Risk of Perinatal HIV * (%)	NBS HIV Screen- Negative and False-Positive	Fisher Exact *p*-Value (α = 0.05)	Odds Ratio	Odds Ratio Range (95% Confidence Interval)	At-Risk Incidence Rate (Per 100,000)
No. specimens	2,614,774	NA	NA	NA	NA	NA	NA	NA
No. newborns	2,306,594	4018	3999	2,302,595	NA	NA	NA	173
Sex (female newborns compared to male newborns)								
Female	1,124,689 (48.8%)	1943 (48.4%)	1932 (48.3%)	1,122,757 (48.8%)	0.54	0.98	0.92–1.04	172
Male	1,178,554 (51.1%)	2073 (51.6%)	2065 (51.6%)	1,176,489 (51.1%)				175
Birth weight (NBW newborns compared to LBW and ELBW newborns)								
Normal birth weight (≥2500 g)	2,122,996 (92.0%)	3340 (83.1%)	3323 (83.1%)	2,119,673 (92.1%)	NA	NA	NA	157
Low birth weight (1500–2499 g)	152,819 (6.6%)	523 (13.0%)	521 (13.0%)	152,298 (6.6%)	<0.001	0.46	0.42–0.50	341
Extremely low birth weight (<1500 g)	28,427 (1.2%)	142 (3.5%)	142 (3.6%)	28,285 (1.2%)	<0.001	0.31	0.26–0.37	500
Gestational Age (term newborns compared to preterm and post-term newborns)								
Term (37–40 weeks, 6 days)	1,903,197 (82.5%)	3147 (78.3%)	3132 (78.3%)	1,900,065 (82.5%)	NA	NA	NA	165
Preterm (<37 weeks)	207,930 (9.0%)	666 (16.6%)	663 (16.6%)	207,267 (9.0%)	<0.001	0.52	0.47–0.56	319
Post-term (≥41 weeks)	188,770 (8.2%)	182 (4.5%)	181 (4.5%)	188,589 (8.2%)	<0.001	2.64	1.48–2.0	96
NICU status (NICU newborns compared to non-NICU newborns)								
NICU	250,395 (10.9%)	975 (24.3%)	971 (24.3%)	249,424 (10.8%)	<0.001	2.64	2.45–2.84	388
Non-NICU	2,056,199 (89.1%)	3043 (75.7%)	3028 (75.7%)	2,053,171 (89.2%)				147
Number of births per pregnancy (single births compared to multiple births)								
Single	2,220,994 (96.3%)	3842 (95.6%)	3823 (95.6%)	2,217,171 (96.3%)	0.025	0.82	0.70–0.96	172
Multiple	83,422 (3.6%)	175 (4.4%)	175 (4.4%)	83,247 (3.6%)				210
Race/Ethnicity ** (Asian newborns compared to each of the other race/ethnicities)								
Total	473,086	929	927	472,159	NA	NA	NA	196
Asian	39,194 (8.3%)	14 (1.5%)	14 (1.5%)	39,180 (8.3%)	NA	NA	NA	36
White	229,087 (48.4%)	147 (15.8%)	146 (15.7%)	228,941 (48.5%)	0.099	0.56	0.299–0.97	64
Black	77,823 (16.5%)	481 (51.8%)	480 (51.8%)	77,343 (16.4%)	<0.001	0.058	0.031–0.098	617
Hispanic	81,451 (17.2%)	191 (20.6%)	191 (20.6%)	81,260 (17.2%)	<0.001	0.15	0.082–0.26	234
Native American	776 (0.2%)	1 (0.1%)	1 (0.1%)	775 (0.2%)	0.51	0.28	0.042–11.7	129
Other/ Unknown	44,755 (9.5%)	95 (10.2%)	95 (10.2%)	44,660 (9.5%)	<0.001	0.17	0.088–0.296	212

* Not including 19 false-positive cases. ** Race/ethnicity data only includes data from 2011 and 2012. Percentages do not always add up to 100% due to missing data or due to rounding to the nearest percent. HIV, human immunodeficiency virus; α, significance value; No., number; NA, not applicable; NBW, normal birth weight; LBW, low birth weight; ELBW, extremely low birth weight; NICU, neonatal intensive care unit.

**Table 2:** Outcomes of newborns referred for HIV follow-up.

Year	Number Referred	Number Infected	Number Infected Per 100,000 Births	Newborn Not Infected. Mother Infected (HIV-1)	Newborn Not Infected. Mother Infected (HIV-2)	Newborn Not Infected. Mother Unknown	Newborn with False-Positive Result	Newborn Expired: No Diagnosis	Newborn Lost to Follow-Up
2011	479	6	2.5	464	2	0	1	1	5
2012	450	1	<1	444	0	1	1	1	2 *
2013	466	2	<1	457	2	0	2	3	0
2014	434	3	1.3	424	0	1	2	1	3
2015	401	0	0	399	0	0	0	0	2
2016	421	2	<1	416	1	0	0	1	1
2017	408	1	<1	402	0	1	2	2	0
2018 **	319	0	0	315	0	0	1	0	3
2022 **	65	0	0	63	0	0	0	0	2
2023	298	0	0	281	0	0	4	0	13
2024	277	1	<1	257	0	0	6	0	13
Total	4018	16	<1	3922	5	3	19	9 ^#^	44 ^ǂ^

* Includes one newborn whose parent declined follow-up. ** For 2018, data from the first 11 months is presented, and for 2022, data from the last 3 months is presented. ^#^ Mothers of all 9 newborns were reported as HIV-positive. ^ǂ^ Mothers of 36 of 44 newborns were reported as HIV-positive. Mothers of the remaining 8 newborns were reported as HIV-negative (*n* = 6), HIV status unknown with expedited testing performed at birth (*n* = 1), and information is missing (*n* = 1). HIV, human immunodeficiency virus.

## Data Availability

Summary data will be readily available; however, the work presented is programmatic data from a screening program, and families have not consented to sharing their newborn’s data. Therefore, individual-level data will not be shared. The NYS NBS Program does not share data from individual newborns.

## Abbreviations

The following abbreviations are used in this manuscript:

ART	Antiretroviral therapy
CDC	Centers for Disease Control and Prevention
CLEP	Clinical Laboratory Evaluation Program
DBS	Dried blood spot
ELBW	Extremely low birth weight
ELISA FDA	Enzyme-linked immunosorbent assay US Food and Drug Administration
HIV	Human immunodeficiency virus
IgG	Immunoglobulin G
IgM	Immunoglobulin M
LBW	Low birth weight
MTCT	Mother-to-child transmission
NAT	Nucleic acid testing
NBS NBW	Newborn screening Normal birth weight
NICU	Neonatal intensive care unit
NYS	New York State
NYC	New York City
PCR	Polymerase chain reaction
RNA	Ribonucleic acid
S/CO	Absorbance to cutoff ratio
SD	Standard deviation
